# Predicting undiscovered non-human primate hosts of Semliki Forest complex Alphaviruses

**DOI:** 10.64898/2026.08.10.26360069

**Published:** 2026-08-11

**Authors:** Michael Celone, Adrian Castellanos, Bernard Okech, Sean Beeman, Simon Pollett, Barbara Han

## Abstract

Arthropod-borne Alphaviruses in the Semliki Forest (SF) virus complex, including Chikungunya virus, Mayaro virus, and O'nyong-nyong virus, represent a substantial threat to human health globally. These antigenically related viruses often cause short-term febrile symptoms that can progress to chronic and debilitating arthropathy. The ecology of these viruses is complex due to the involvement of various animal hosts and mosquito vectors in their transmission cycles. Non-human primates (NHPs) have been identified as potentially important animal hosts that may contribute to ongoing transmission and emergence, but the full range of known NHP hosts is not clear. Due to the epidemiological importance of NHPs, we predicted NHP species with a high probability of being carriers of SF complex Alphaviruses. We first compiled an extensive database of intrinsic and extrinsic NHP traits including reproduction, diet, behavior, biogeography, home-range, and climate. Next, we identified NHP species that are known zoonotic hosts of SF complex Alphaviruses. Hosts are defined as naturally infected NHPs identified through field studies. They do not necessarily meet the criteria for reservoir competence. Host vs. non-host status was largely determined through serology and species without data were treated as non-hosts in our analysis. Finally, we used boosted regression trees (BRT) to develop a trait profile of the known NHP host species. Using this trait profile, we identified additional, potentially unrecognized NHP hosts with a comparable trait profile. We found that latitudinal range, maximum longevity, maximum temperature, minimum human population density, number of ecoregions in species range, neonate mass, female mass, and mean precipitation were important predictors of zoonotic host status. Additionally, we were able to distinguish NHP hosts from non-hosts, and to identify 30 additional NHP species predicted to carry SF complex Alphaviruses. These findings can serve as hypotheses that can guide targeted surveillance and may help direct additional field epidemiological studies to better define the risk and risk factors of Alphavirus emergence.

## Full Text Availability

The license terms selected by the author(s) for this preprint version do not permit archiving in PMC. The full text is available from the preprint server.

